# Segregate or cooperate- a study of the interaction between two species of *Dictyostelium*

**DOI:** 10.1186/1471-2148-8-293

**Published:** 2008-10-24

**Authors:** Chandra N Jack, Julia G Ridgeway, Natasha J Mehdiabadi, Emily I Jones, Tracy A Edwards, David C Queller, Joan E Strassmann

**Affiliations:** 1 Department of Ecology and Evolutionary Biology, Rice University, Houston, TX, USA; 2 Smithsonian Institution, National Museum of Natural History, PO Box 37012, Washington, DC 20013, USA; 3 Department of Ecology and Evolutionary Biology, The University of Arizona, Tucson, AZ, USA

## Abstract

**Background:**

A major challenge for evolutionary biology is explaining altruism, particularly when it involves death of one party and occurs across species. Chimeric fruiting bodies of *Dictyostelium discoideum *and *Dictyostelium purpureum *develop from formerly independent amoebae, and some die to help others. Here we examine co-aggregation between *D. discoideum *and *D. purpureum*, determine its frequency and which party benefits, and the extent of fair play in contribution to the altruistic caste.

**Results:**

We mixed cells from both species in equal proportions, and then we analyzed 198 individual fruiting bodies, which always had either a *D. discoideum *or *D. purpureum *phenotype (*D. discoideum*- 98, *D. purpureum*- 100). Fifty percent of the fruiting bodies that looked like *D. discoideum *and 22% of the fruiting bodies that looked like *D. purpureum *were chimeric, though the majority of spores in any given fruiting body belonged to one species (*D. discoideum *fruiting bodies- 0.85 ± 0.03, *D. purpureum *fruiting bodies- 0.94 ± 0.02). Clearly, there is species level recognition occurring that keeps the cells mostly separate. The number of fruiting bodies produced with the *D. discoideum *phenotype increased from 225 ± 32 fruiting bodies when *D. discoideum *was alone to 486 ± 61 in the mix treatments. However, the number of *D. discoideum *spores decreased, although not significantly, from 2.75e^7 ^± 1.29e^7 ^spores in the controls to 2.06e^7 ^± 8.33e^6 ^spores in the mix treatments. *D. purpureum *fruiting body and spore production decreased from 719 ± 111 fruiting bodies and 5.81e^7 ^± 1.26e^7 ^spores in the controls to 394 ± 111 fruiting bodies and 9.75e^6 ^± 2.25e^6 ^spores in the mix treatments.

**Conclusion:**

Both species appear to favor clonality but can cooperate with each other to produce fruiting bodies. Cooperating amoebae are able to make larger fruiting bodies, which are advantageous for migration and dispersal, but both species here suffer a cost in producing fewer spores per fruiting body.

## Background

Cooperative relationships between different species are common in nature [1-3]. They can be found in every environment, from cactus pollinators in the desert [4] to microbial symbionts in the ocean [5]. The evolution of cooperation presents a conundrum. How do these relationships evolve and remain stable over generations? Why would selection favor altruism and cooperation when cheaters could reap the benefits of an interaction without paying any of the associated costs [6-9]?

Only in recent years have the worlds of microbiologists and evolutionary biologists merged to begin interdisciplinary studies on cooperation in microorganisms [10,11]. The social amoebae of the genus *Dictyostelium *present ideal candidates for studying microbial interactions. The first species, *D. mucoroides *Brefeld, was isolated from horse dung more than a century ago. Today approximately one hundred species have been formally described [12]. In 1984, Raper set up a comprehensive taxonomic system that classified all species based on morphological differences such as the presence or absence of polar spore granules, sorus color, stalk features, and overall size [13]. Recently, Schaap *et al. *derived a phylogenetic tree of almost every described species using SSU rDNA gene sequences that now allows more exact species identification [14].

**Figure 1:**
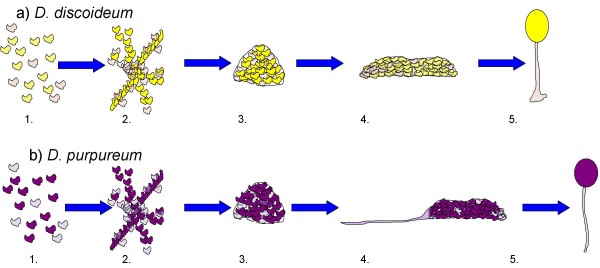
**The life cycle of two *Dictyostelium *species**. Both species have a similar developmental lifecycle until the slug stage. The darker cells are the spore cells and the lighter cells are the stalk cells. 1) The cells eat bacteria and reproduce asexually. 2) Upon starvation, the cells begin to aggregate together using cyclic AMP as a chemo-attractant. 3) In late aggregation, the cells form a mound. 4) The cells form a multicellular slug. In *D. discoideum*, the slug migrates to a new location, forms a stalk, and then completes development. In *D. purpureum*, the slug forms a stalk as it migrates to a new location and then completes development. 5) The final fruiting body stage where some of the cells have become sterile to form a stalk and hold up the reproductive spore body.

While there are some differences in behavior, most species follow the same lifecycle. All dictyostelid species spend the majority of their lifecycle as solitary amoebae living on the forest floor, eating bacteria. When the cells begin to starve, they send out a signal, which is cyclic AMP in many species, causing all nearby cells to aggregate together. In some species, such as *D. discoideum *(Figure 1A), the cells form a multi-cellular slug that then migrates to a new location. Once migration is complete, approximately one-fifth of the cells will altruistically die to form a sterile stalk to hold aloft the remaining cells, which have formed a sorus consisting of viable spores [13,15]. In other species, including *D. purpureum *(Figure 1B), the slug forms a sterile stalk as it migrates to a new location. Once migration is complete, the stalk becomes vertical and the sorus forms. At this stage in the lifecycle of *Dictyostelium *conflict may occur as the amoebae make the transition to multicellularity from individual cells. Some of the cells undergo the ultimate sacrifice of dying to form the sterile stalk, leaving the majority to form fertile spores. This altruistic behavior will be favored by natural selection only if those cells are able to pass on their genes through relatives, who may often be clone mates. The higher the relatedness to spore cells, and the greater the advantage to having a stalk, the more the stalk cells will benefit from paying the cost and the less conflict there will be between the two cell types. The social behavior of *Dictyostelium discoideum *makes it an ideal model to study social evolution [16]. It has been shown, both in the lab and in nature, that *D. discoideum *clones will form chimeras [16-19]. In these chimeric fruiting bodies, conflict can occur. Foster *et al. *[17] reported that chimeric slugs migrate less far than clonal slugs of the same size, indicating that some form of conflict between the clones is occurring within the slug. Additionally, some clones of *D. discoideum *have the ability to cheat other *D. discoideum *clones in chimeras by forcing them into the stalk, leading to an unequal representation in the fruiting body [16]. Recently it has been shown that clones of two other species of social amoeba, *D. purpureum *and *D. giganteum *form intraspecific chimeras [18,19].

A single soil sample of a fifth of a gram may contain several clones and species [20]. Yet, the formation of interspecific chimeras has not been carefully studied even though many of these species aggregate to the same chemo-attractant, cyclic AMP. Olive [21] first looked for and failed to find chimeras of *D. purpureum *and *D. mucoroides*, followed by two other groups almost fifty years later [22,23]. Neither group managed to find chimeras under normal aggregation conditions. Another researcher, Hagiwara [24], made some preliminary interspecific mixtures while he explored whether aggregating streams of cells of three different genera of Dictyostelids mixed or overlapped in any way. His work verified that many species use the same chemo-attractants.

A molecular phylogeny of the Dictyostelids based on small subunit RNA and α-tubulin sequences shows subdivision of all known species into four major groups. *D. discoideum*, *D. purpureum*, and *D. giganteum *are all members of Group 4, a group where all of the studied species aggregate and respond to the same chemo-attractant, cAMP [14]. Most of the studies exploring social behavior in the Dictyostelids have concentrated on within-species interactions in *D. discoideum*. Although they are found in the same group and have similar biological properties, *D. discoideum *and *D. purpureum *are not even close sister species. Ninety percent of the homologs between *D. discoideum *and *D. purpureum *have less than 75% identity and over 60% of the homologs have diverged so long ago that dS cannot be calculated due to synonymous site saturation [Xiangjun Tian, pers. comm.]. However, species interactions between members of Group 4 have not been closely studied, even though these species are often found together in nature. Here we test the hypothesis that *D. discoideum *and *D. purpureum *form chimeric fruiting bodies when mixed together and we examine the affect of this interaction on both spore production and fruiting body production.

## Results

### Chimerism of *D. discoideum *and *D. purpureum *fruiting bodies

We found that more than 30% of the fruiting bodies we examined were chimeric in 20 out of 21 trials where the initial cell suspension contained an equal number of cells of both species. All fruiting bodies in each experiment displayed either the *D. discoideum *phenotype or the *D. purpureum *phenotype. None of the fruiting bodies, including those that were chimeric, displayed an intermediate phenotype.

Forty-nine of the 98 *D. discoideum *fruiting bodies examined contained spores of *D. purpureum*, while only 22 of the 100 *D. purpureum *fruiting bodies contained *D. discoideum *spores (W_14,13 _= 124, n = 27, p < 0.05).

**Figure 2:**
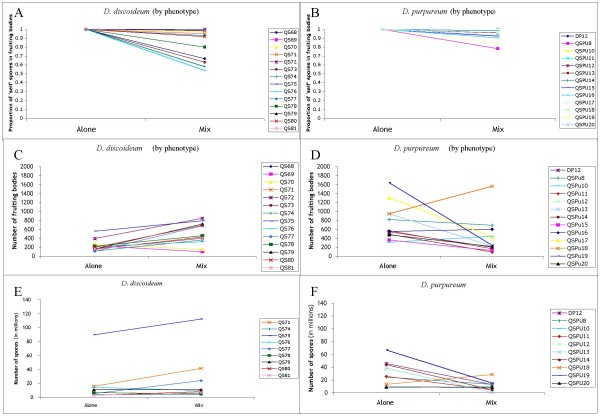
**The effects of the interaction of *D. discoideum *and *D. purpureum***. The graphs show the results of analyses of *D. discoideum *and *D. purpureum *by clone, both when alone and when mixed with each other. A-B: The composition of the fruiting bodies of each species, alone and mixed, where the higher the percentage means the more clonal the fruiting body. C-D: Fruiting body production of each species, alone and when mixed, after being standardized for the number of cells of each species added to a plate. E-F: The number of spores produced by each species, alone and mixed after being standardized for the number of cells of each species added to a plate.

The majority species in the chimeras, with a few exceptions, determined the phenotype. Chimeric *D. discoideum *fruiting bodies contained an average of 26.8 ± 4.4% *D. purpureum *spores per clone while chimeric *D. purpureum *fruiting bodies had 29.5 ± 9.2% *D. discoideum *spores (W_12,8 _= 51, n = 20, p = 0.851). For the exceptions, in 20% of the chimeric fruiting bodies examined in both species, the minority species determined the phenotype. In those *D. discoideum *chimeras the average composition of the fruiting bodies was 82.7 ± 4.9% *D. purpureum *spores but 4 of the 10 fruiting bodies showed only *D. purpureum *spores. In those *D. purpureum *chimeras the average composition of the fruiting bodies was 73.1 ± 9.6% *D. discoideum *spores and only 1 of the 5 fruiting bodies showed only *D. discoideum *spores. However, when we include all fruiting bodies, chimeric and clonal, from the experimental plates, fruiting bodies with the *D. discoideum *phenotype contained a higher percentage of 'nonself' spores, than *D. purpureum *fruiting bodies, although it was not significant. (DD_nonself spores _= 16.04 ± 4.52%, DP_nonself spores _= 5.44 ± 1.90%, W_14,13 _= 125, n = 27, p = 0.101, Figures 2A–B).

### Numbers of fruiting bodies with the morphology of *D. discoideum *vs. *D. purpureum*

We counted the number of fruiting bodies produced by each species on the control plates (each species alone) and on the experimental plates (50:50 mix of the two species) to compare the number of fruiting bodies produced after we standardized for the difference in cell number. We distinguished the fruiting bodies based solely on phenotype and not on whether the fruiting bodies may have contained spores of the other species. In the controls, *D. discoideum *produced 225 ± 32 fruiting bodies per 2 × 10^7 ^cells, while *D. purpureum *produced an average of 719 ± 111 fruiting bodies per 2 × 10^7 ^cells, (W_14,15 _= 10, n = 27, p < 0.001).

The number of fruiting bodies with *D. discoideum *morphology significantly increased to 486 ± 61 when plated with *D. purpureum *when compared to the number of fruiting bodies produced when alone (W_14,13 _= 35, n = 27, p < 0.01, Figure 2C). Conversely, the number of fruiting bodies with *D. purpureum *morphology decreased significantly to 394 ± 111 when plated with *D. discoideum *(W_13,13 _= 135, n = 26, p < 0.01, Figure 2D).

### Spore production by *D. discoideum *and *D. purpureum *from cells

We determined the number of spores produced by each species after equal numbers of cells of each species were mixed together without food to determine if one species gained an advantage over the other. We corrected for germination efficiency and initial cell number when we compared control plates to experimental plates. *D. discoideum *produced the same number of spores from a given number of cells whether or not cells of *D. purpureum *were also present. (DD_spores_exp _= 2.06e^7 ^± 8.33e^6^, DD_spores_ctrl _= 2.75e^7 ^± 1.29e^7^, W_9,9 _= 29, n = 18, p = 0.331, Figure 2E). However, *D. purpureum *produced fewer spores when cells of *D. discoideum *were present compared to when *D. purpureum *cells were alone (DP_spores_exp _= 9.75e^6 ^± 2.25e^6^, DP_spores_ctrl _= 5.81e^7 ^± 1.26e^7^, W_10,10 _= 81.5, n = 20, p = < 0.05, Figure 2F). We also calculated the number of spores produced per fruiting body for both *D. discoideum *and *D. purpureum *when alone and when mixed with each other as a measure of fruiting body size. Both *D. discoideum *and *D. purpureum *produced fewer spores per fruiting body in mixes when compared to the number produced when alone but neither was significant (DD_exp _= 4.16e^4 ^± 1.54e^4^, DD_ctrl _= 6.15e^4 ^± 1.55e^4^, W_9,9 _= 60, n = 18, p = 0.094, DP_exp _= 3.65e^4 ^± 6.74e^3^, DP_ctrl _= 4.96e^4 ^± 6.74e^3^, W_10,10 _= 53, n = 20, p = 0.853).

### Relatedness

We defined relatedness (r) as the probability that two spores in the same fruiting body were from the same species. Relatedness was calculated in the experimental fruiting bodies using p^2 ^+ (1-p)^2 ^where p was the proportion of *D. purpureum *spores in a fruiting body. This measures the degree to which clones experience their own type in the fruiting body, above the population expectation of near zero. Fruiting bodies with the *D. purpureum *phenotype had an average relatedness of r = 0.943 ± 0.014, which was not significantly higher than the relatedness of those fruiting bodies with a *D. discoideum *phenotype, r = 0.89 ± 0.022 (W_14,13 _= 60.5, n = 27, p = 0.140).

### Time-lapse microscopy

**Figure 3:**
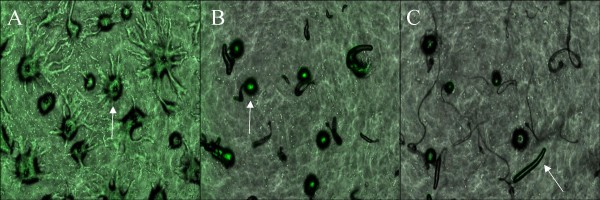
**Slugs of *D. discoideum *and *D. purpureum *show partial sorting due to development difference**. Although cells of both species aggregate together (A), the majority of cells of *D. purpureum *forms a slug first and migrates away, taking some *D. discoideum *cells (indicated by the green) with it (B). The majority of the cells of *D. discoideum *stays in the mound to later form a slug and migrate (C).

We used time-lapse microscopy to determine when the cells of *D. discoideum *and *D. purpureum *aggregate together and when they begin to sort. We observed the different stages of development in one pair, (Experiment #4: QS71 and QSPu16) by labeling QS71 with CellTracker™ Green CMFDA. We found that the cells aggregate through the mound stage, the point in the life cycle after aggregation when the cells are found in small mounds before they differentiate into slugs, and then a primarily *D. purpureum *slug migrates away, leaving behind a mound composed mostly of *D. discoideum *cells that eventually become a fruiting body (Figure 3).

## Discussion

More than one-third of the fruiting bodies we examined were chimeric and 95% of the experiments contained at least one chimeric fruiting body. This shows that, at least in a lab setting, *D. discoideum *and *D. purpureum *cells can interact, aggregate, and form chimeric fruiting bodies, although the average percentage of one clone in any given fruiting body is 90%, which indicates that the two species prefer to segregate but do so imperfectly. Both species had an equivalent proportion of the other species in the chimeric fruiting bodies, but because chimeras were more frequent in *D. discoideum*, there were more foreign spores found in fruiting bodies with the *D. discoideum *morphology. We measured relatedness to determine how much mixing and sorting is happening between the two species. The higher the relatedness, the less intermixing is occurring between the two species. An r-value of 0.5 means that the cells are randomly mixing, while an r-value of 1 means that the cells are completely sorting. Despite the presence of chimeric fruiting bodies, relatedness remained high within fruiting bodies of each phenotype (0.89 for *D. discoideum*, 0.94 for *D. purpureum*) on plates that began with an equal number of cells of each species.

It is remarkable, for several reasons, that we found such a high incidence of chimerism First, *D. discoideum *and *D. purpureum *are not particularly closely related. In the current phylogeny [14], the node separating the two species has 4 other species in the branch including *D. discoideum *and 17 others in the branch including *D. purpureum*. This phylogenetic distance is manifest in several developmental differences between the two species that may impact the level of sorting. *D. purpureum *forms a stalk as the slug migrates, while *D. discoideum *forms its stalk after the slug finishes migrating [13]. Additionally, *D. purpureum *develops faster than *D. discoideum. *As a result, cells of *D. purpureum *may differentiate first leading to an increase in sorting if the genes responsible for cell-type partitioning and development up-regulate at different times. We found evidence of this pattern when we used time-lapse microscopy. Cells of both species aggregate together for a short time, but then *D. purpureum *slugs break off and migrate away from the initial mound, leaving mostly *D. discoideum *cells. A short time later, *D. discoideum *slugs begin to migrate and then form fruiting bodies. Interestingly, slugs contained cells from both species, indicating only partial disassociation.

Our chimerism result is also surprising because prior research failed to show chimerism, despite having mixed different species in a variety of ways. Raper and Thom [22] first mixed spores of *D. discoideum *and *D. purpureum *and reported the absence of intermediate phenotypes, which was in accordance with our results, but does not preclude chimerism. They then mixed *D. discoideum *spores with spores of *D. mucoroides*, a species that is as equally distant phylogentically as *D. purpureum *[14]. They used the bacterium *S. marcescens *as a food source. *S. marcescens *contains a red pigment that *D. discoideum *is unable to digest, resulting in dyed cells [13,25] while *D. mucoroides *digests the pigment and remains white. They found that the red cells initially aggregated together with the white cells but separated into red and white fruiting bodies. This shows that most cells segregated, but it is not clear if some individual cells of the wrong type might have been present.

Raper and Thom [22] also tried making grafts between different portions of the slugs of *D. discoideum *and *D. purpureum*, but were unsuccessful in getting the segments to permanently coalesce and form chimeric fruiting bodies. Using these data, they concluded that *Dictyostelium *species did not form chimeras. In one final experiment, they were able to obtain fruiting bodies with intermediate phenotypes by allowing cells of each species to form slugs and then crushing those slugs and mixing them [22]. These fruiting bodies contained spores from both species. However, those fruiting bodies that retained the phenotype of only one parent only produced fruiting bodies of that same phenotype, seemingly indicating that those fruiting bodies consisted of one species. Bonner and Adams [23] also failed to find chimeras after they completed a series of experiments where they attempted to make intermediate fruiting bodies by grafting different species together during the aggregation stage. Neither group reported the density of spores that they used.

Perhaps we were able to find chimeras while the others did not because we plated out individual spores from fruiting bodies carefully at a very low density so we could detect low levels of mixing. Overall, there was mostly sorting, but there was some mixing, which may have been missed if not looked for carefully. We also used multiple clones, and had we used only one pair, an unlucky choice (for example mix 10 between clones QS75 and QSPu13 in Figure 2A–B) could have led us to the false conclusion that there was little mixing.

Finally, the finding of chimerism between species is surprising because both species apparently avoid chimerism even with other clones of their own species. Gilbert *et al. *[26] found that the relatedness for naturally occurring fruiting bodies collected in the wild that contained multiple clones of *D. discoideum *was 0.68, which was much lower than the overall relatedness of 0.98, because there were many clonal fruiting bodies. This result could be due either to sorting or to patchy distribution of clones. However, clear sorting was shown in fruiting bodies of *D. purpureum *when pairs of clones were mixed in 50:50 ratios; the result was an overall relatedness of 0.81 [18]. Recently, somewhat weaker sorting has also been demonstrated between *D. discoideum *clones (Ostrowski *et al. *submitted). Our relatedness values for the two species mixed 50:50, were 0.89 for *D. discoideum *and 0.94 for *D. purpureum. *The higher values indicate greater clonal sorting than within-species mixes.

Why do these two species cooperate at least some of the time and is it true mutualism? Our system is unique and interesting in that it defies previous explanations of mutualism. In most cooperative interactions involving different species, each partner brings different goods or services to the association, such as between the Senita cactus and Senita moth, where the moth pollinates the cactus in exchange for a place to oviposit eggs and the larvae to subsequently eat a portion of the seeds [4]. That is not the case with these two Dictyostelids because both species provide essentially the same services – migration and stalk formation. Mutualisms are now being recognized as lying on a continuum with parasitism. Some people also hypothesize that mutualism evolved from parasitism and that mutualism is best described as mutual exploitation [27,28]. It may be that the two species are exploiting each other differentially, with *D. discoideum *benefiting most in the metric we measured. If this true, it may be that this interaction lies on the boundary between the two and is heading towards mutualism.

It is possible that the mixing is a mistake. Each species may undergo its social lifecycle where certain cells altruistically form stalk cells as it would if in a clonal population. Cells of different species may aggregate and develop together because of their close proximity to each other and similar developmental characteristics. Another possibility is that although this interaction evolved to provide beneficial cooperation within species (or even within clones), different species are able to benefit from those services, such as protection from predators, migration, spore formation and dispersal when they would otherwise not be able to because of a cell number deficiency. When both species face the possibility of being unable to aggregate on their own because they lack sufficient cell number, the two species will aggregate together and form fruiting bodies, to their mutual benefit, instead of dying out. Though we are unable to fully distinguish these hypotheses, we can provide an accounting of some of the costs and benefits that result from interspecies chimerism.

In any cooperative relationship, there are costs associated with each altruistic act. One such cost is that the altruistic act is not reciprocated, which may lead to the exploitation of one partner by the other. Cheating is the greatest concern when there is an interaction between two individuals that are not genetically identical. Earlier research shows that clones of *D. discoideum *may cheat each other, but prior experiments involving only *D. purpureum *clones show that the species maintains a high degree of kin discrimination by preferentially associating with kin without displaying a consistent pattern of cheating [16,18]. The stronger segregation seen in *D. purpureum *may have evolved as a way to prevent cheating between clones, but it also might mean that this species no longer has a need to maintain mechanisms of cheating, or other defenses against cheating. When the two species are mixed together, *D. discoideum*'s ability to cheat and *D. purpureum*'s lack of a cheating mechanism may be the reason *D. purpureum *was exploited.

It is possible that this association is kept stable and that cheating is kept to a minimum because the aggregates form only when necessary and that they are kept as pure as possible, as indicated by the much higher relatedness values we calculated when compared to those found in previous studies. Additionally, both species suffered in the production of spores per fruiting body, which may be why the two species tend to segregate from each other despite some of the benefits that may be gained from the interaction.

We did not observe a clear benefit to this interaction that might explain why it has persisted. In terms of spore production, *D. discoideum *maintained the number of spores it produces while *D. purpureum *decreased the number of spores produced. However, additional possible benefits result from larger slug sizes that are not measurable using spore production, the metric we tested. One possible benefit for cells from both species is protection from predators. By aggregating together, the amoebae can initiate mechanisms to avoid soil predators such as nematodes. Kessin *et al. *[29] showed that *Caenorhabditis elegans *feeds on individual amoebae up through early aggregation. However, in late aggregation the cells form a polysaccharide sheath that the nematodes are unable to penetrate. This sheath protects the amoebae as they migrate as a multicellular slug. Once the fruiting body is formed, *C. elegans *may ingest the spores, but they are unable to digest them. Kessin *et al. *[29] found an additional benefit in *D. purpureum*: at high cell densities, it is able to repel nematodes. Therefore, it may be beneficial to both species to aggregate together when cell numbers are low, especially in the presence of predators.

Migration distance is another potential benefit of forming a larger slug. Foster *et al. *[17] found that larger slugs of *D. discoideum *traveled further than slugs containing half the number of cells. Also, they found that larger chimeric slugs traveled further than smaller clonal slugs. When slugs are traveling to a new location because the current one has run out of bacteria, larger slugs are more likely, over both smaller slugs and solitary cells, to reach a new patch of bacteria [30,31].

A final possible benefit to co-aggregation is for spore dispersal purposes. To successfully disperse spores, they must be held aloft on a stalk of sufficient height. If there are too few cells in the aggregate, a fruiting body may not form at all. Or, even if a small fruiting body is able to form, it may be at a disadvantage relative to larger fruiting bodies, making it less likely to disperse due to contact from passing invertebrates.

Although we can only hypothesize about possible benefits to both species from cooperating, a mutualism would not evolve between these two species without gaining some type of fitness benefit. In single species fruiting bodies, some cells altruistically give up reproduction so that equally related cells become reproductive spores. In our experiments, cells still forfeit their reproductive ability so that related cells benefit. However, cells of the other species also benefit through by-product altruism, as they too are able to form reproductive spores because of the sacrifice of the other cells.

## Conclusion

The surprising finding that *D. discoideum *and *D. purpureum *can cooperate to form chimeric fruiting bodies cannot be explained by increased spore production. It may simply be a mistake or it may be making the best of a bad job. Both species seem to favor being clonal, but a fraction of cells cooperate with the other species, perhaps when benefits are high enough to overcome the cost in decreased spore production. Cooperating amoebae are able to make larger fruiting bodies, which are advantageous for migration and dispersal, but these benefits will need to be quantified to assess their importance.

## Methods

### Clones

We used fourteen genetically distinct wild clones of *D. discoideum *and thirteen clones of *D. purpureum *isolated from different soil samples collected at the Houston Arboretum, Texas (Table 1).

**Table 1:** Table of *D. discoideum *and *D. purpureum *clones by experiment

EXPERIMENT	D. discoideum	D. purpureum
1	QS68	QSPu16
2	QS69	QSPu16
3	QS70	QSPu16
4	QS71	QSPu16
5	QS72	QSPu16
6	QS73	QSPu15
7	QS73	QSPu16
8	QS73	QSPu17
9	QS74	QSpu18
10	QS75	QSPu13
11	QS76	DP12
12	QS71	QSPu14
13	QS77	QSPu8
14	QS77	QSPu19
15	QS78	QSPu10
16	QS78	QSPu11
17	QS79	QSPu20
18	QS78	QSPu20
19	QS80	QSPu20
20	QS81	QSPu12
21	QS81	QSPu14

### Cell preparation

We plated out 3 × 10^5 ^spores from each clone with 300 μl of the bacteria *Klebsiella aerogenes *(KA) as food on SM/5 agar plates [32]. After approximately 38 hours, we harvested the cells while they were in log growth before multi-cellular development occurred with cold standard KK2 buffer (3.8 mM K_2_HPO_4_, 16.5 mM KH_2_PO_4_). The cells were then centrifuged three times at 1000 rpm for three minutes to remove any remaining bacteria and set at a concentration of 10^8 ^cells per milliliter in KK2 buffer.

### Experiment set-up

For each of the 21 experiments, we tested one *D. discoideum *clone with one *D. purpureum *clone. We filled each well of a 6-well tissue culture plates (3.5 cm in diameter) with 7 ml of non-nutrient agar (14.9 g agar per liter KK2 buffer). We designated four of the wells, from here forward called plates, as control plates. We labeled the final two plates as experimental plates. For the control plates, we added 4 × 10^7 ^cells in 400 μl of KK2 buffer for each clone. For the experimental plates, we added together 2 × 10^7 ^cells of each clone in 200 μl of KK2 buffer. After thoroughly mixing the cells, we spread 400 μl of the cell suspension on a plate. Thus, we had a replicate of the control and experimental plates. We used one set to assess mixing and the other to assess spore production. We used both sets to assess fruiting body production.

### Data collection and analyses

#### Fruiting body assessment

**Figure 4:**
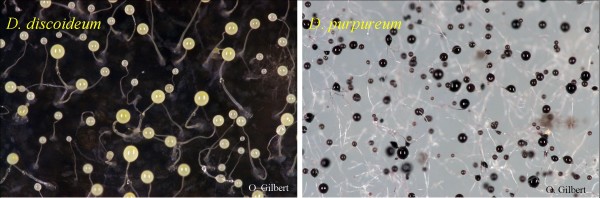
**Characteristics used to determine *Dictyostelium *phenotype**. *D. discoideum *is characterized by having a basal disc; thick, straight stalk, and white/clear sorus. *D. purpureum *is characterized by its lack of basal disc; thin, wavy stalk; and purple sorus.

In order to determine the number of fruiting bodies present on all plates, we created a circular grid 3.5 cm in diameter that exactly fit the bottom of the tissue culture plates. Each square in the grid had an area of 0.25 cm^2^. Before the start of the experiment we randomly selected eight of the squares to be the counting squares so that the same squares were used consistently for all 21 of the experiments. In these squares, we counted all of the fruiting bodies, and in the case of the experimental plates, whether they had a *D. discoideum *or *D. purpureum *phenotype using a set of established criteria such as sorus color, presence of a basal disc, and stalk type (Figure 4). We examined the remaining squares for the presence or absence of fruiting bodies of each species. We then calculated the number of fruiting bodies by multiplying the average number of fruiting bodies over the eight squares by the area of the plate.

#### Spore production assessment

For one of each control and experiment plate, we collected all of the fruiting bodies in 1 ml of KK2 buffer to count the number of spores that were produced. We used a hemacytometer to count the spores. Additionally, we plated out a dilute sample of the spores from the experiment on five 60 cm Petri plates containing SM/5 agar to determine the proportion of spores produced for each species. By plating a diluted concentration of the spores, we were able to determine where on the plate cells were released from individual spores and were then able to determine the identity of the spore and calculate the proportion of spores of each species. After adjusting for germination efficiency, we were able to determine the number of spores produced by each species on the experimental plates by multiplying the proportion of spores of each species previously calculated by the total number of spores that were collected from the experimental plate.

#### Germination efficiency

For each clone, we plated out approximately 30 spores per plate over six 60 cm Petri plates containing SM/5 agar with 300 ul of KA. After three days, we began scoring the plates for germinated spores, indicated by clearings in the bacteria. We replicated this procedure twice to get an average number of spores that germinate for each species. We also plated out an equal known number of spores from both species together at low density to see if the spores of one species inhibited the other and prevented them from germinating.

When plated at low density, the average germination rate for *D. discoideum *was 17.7% (SE = 0.022) while the average rate of germination for *D. purpureum *was 50.3% (SE = 0.033) (F_1,58 _= 59.41, n = 60, p < 0.001). We also tested the germination efficiency of each species alone and when plated with the other species to ensure that the spores of one species were not inhibiting spores of the other. We found that there was no difference in the germination efficiency for either *D. discoideum *(F_1,16 _= 0.31, n = 18, p = 0.585) or *D. purpureum *((F_1,15 _= 0.31, n = 17, p = 0.361) in mixes as compared to pure clones. Based on these results, we adjusted spore numbers to reflect the greater spore germination rate of *D. purpureum*.

#### Chimera assessment

From the other set of experiment plates, we collected five fruiting bodies that had a *D. discoideum *phenotype and five fruiting bodies that had a *D. purpureum *phenotype. We placed each fruiting body individually in 40 μl of KK2 buffer and plated out a dilute sample of the spores with on SM/5 plates with KA. We tallied the number of spores of each species that hatched from the fruiting bodies to determine if the fruiting body was chimeric and what percentage of the spores in the fruiting body were of the other species' phenotype after again adjusting for germination.

#### Timelapse Florescence Microscopy

We observed the different stages of development in one pair, (Experiment #4: QS71 and QSPu16) by labeling QS71 with CellTracker™ Green CMFDA. We followed the manufacturer's recommended protocol to label the cells, except that we used 50 μM of CellTracker™ Green CMFDA. We created the timelapse using a Nikon™ E1000 florescent microscope and MetaMorph^® ^imaging software.

#### Analyses

We ran Wilcoxin rank sum tests on all our data except the germination efficiency results. The data were analyzed after grouping by clone, although when the data were grouped by experiment, the results were comparable. The germination efficiency data was analyzed using ANOVAs. We ran all analyses on our data using R [33]. All of the data are reported as the mean ± standard error. The graphs were created using Microsoft Excel version 11.3.5.

## Authors' contributions

CNJ, DCQ, JES, and NJM designed and conducted the research, analyzed the data, and wrote the manuscript. CNJ, DCQ, JES, NJM, and EIJ designed and conducted research on the initial experiment. CNJ, NJM, JGR, and TAE conducted the experiments. All authors read and approved the final manuscript.
